# Natural Type 3/Type 2 Intertypic Vaccine-Related Poliovirus Recombinants with the First Crossover Sites within the VP1 Capsid Coding Region

**DOI:** 10.1371/journal.pone.0015300

**Published:** 2010-12-21

**Authors:** Yong Zhang, Shuangli Zhu, Dongmei Yan, Guiyan Liu, Ruyin Bai, Dongyan Wang, Li Chen, Hui Zhu, Hongqiu An, Olen Kew, Wenbo Xu

**Affiliations:** 1 WHO WPRO Regional Polio Reference Laboratory and State Key Laboratory for Molecular Virology and Genetic Engineering, National Institute for Viral Disease Control and Prevention, Chinese Center for Disease Control and Prevention, Beijing, People's Republic of China; 2 Taishan Medical University, Taishan City, People's Republic of China; 3 Division of Viral Diseases, National Center for Immunization and Respiratory Diseases, Centers for Disease Control and Prevention, Atlanta, Georgia, United States of America; University of Hong Kong, Hong Kong

## Abstract

**Background:**

Ten uncommon natural type 3/type 2 intertypic poliovirus recombinants were isolated from stool specimens from nine acute flaccid paralysis case patients and one healthy vaccinee in China from 2001 to 2008.

**Principal Findings:**

Complete genomic sequences revealed their vaccine-related genomic features and showed that their first crossover sites were randomly distributed in the 3′ end of the *VP1* coding region. The length of donor Sabin 2 sequences ranged from 55 to 136 nucleotides, which is the longest donor sequence reported in the literature for this type of poliovirus recombination. The recombination resulted in the introduction of Sabin 2 neutralizing antigenic site 3a (NAg3a) into a Sabin 3 genomic background in the *VP1* coding region, which may have been altered by some of the type 3-specific antigenic properties, but had not acquired any type 2-specific characterizations. NAg3a of the Sabin 3 strain seems atypical; other wild-type poliovirus isolates that have circulated in recent years have sequences of NAg3a more like the Sabin 2 strain.

**Conclusions:**

10 natural type 3/type 2 intertypic *VP1* capsid-recombinant polioviruses, in which the first crossover sites were found to be in the *VP1* coding region, were isolated and characterized. In spite of the complete replacement of NAg3a by type 2-specific amino acids, the serotypes of the recombinants were not altered, and they were totally neutralized by polyclonal type 3 antisera but not at all by type 2 antisera. It is possible that recent type 3 wild poliovirus isolates may be a recombinant having NAg3a sequences derived from another strain during between 1967 and 1980, and the type 3/type 2 recombination events in the 3′ end of the *VP1* coding region may result in a higher fitness.

## Introduction

Polioviruses, the causative agents of acute paralytic poliomyelitis, have three serotypes and are members of the human enterovirus C species of *Enterovirus* genus in the *Picornaviridae* family [1]. Polioviruses are small, nonenveloped human enteroviruses in which the virion consists of 60 copies of each of four capsid proteins (VP4 to VP1) surrounding a 7,500 nucleotide (nt) positive-sense, single-stranded polyadenylated RNA genome. The viral RNA contains a long, open reading frame flanked by a 5′-untranslated region (UTR) and a 3′-UTR. A single polyprotein translated from the RNA strand is first cleaved into three polyprotein precursors: P1, P2, and P3. P1 is processed to yield four capsid proteins: VP4, VP2, VP3, and VP1. P2 and P3 are the precursors of nonstructural proteins: 2A to 2C and 3A to 3D [2].

The trivalent oral polio vaccine (OPV) contains three different poliovirus serotypes (type 1, 2, and 3). The use of OPV permits coinfection of the human gut cells with type 1, type 2, and type 3 vaccine strains, and thus providing favorable conditions for intertypic recombination. In fact, recombination is a very frequent phenomenon in poliovirus evolution and has been frequently found in patients with vaccine-associated paralytic poliomyelitis (VAPP) [3], [4], [5]. Although OPV is safe, it can circulate silently in the population with low vaccine coverage for a few months and then revert from an attenuating pattern to a neurovirulent one (vaccine-derived polioviruses, VDPVs) to cause an outbreak [6], [7], [8], [9], [10], [11]. Circulating VDPVs (cVDPVs) represent strains that show ≤99% *VP1* coding region sequence homology to the ancestral Sabin strains and can cause sustained person to person transmission [11], [12]. Most of the cVDPVs strains, except Chinese cVDPVs strains [10], have evolved capsid-region sequences as well as unidentified recombinant noncapsid sequences; these sequences are thought to be derived from human enterovirus C species by recombination [6], [7], [8], [9]. Two genetic characteristics, nucleotide mutations at key neurovirulence determination sites and genetic rearrangements with human enterovirus C species, seem to underlie the occurrence of poliomyelitis outbreaks associated with cVDPVs [7], [12], [13].

While the genetic variability of polioviruses is mostly due to nucleotide substitutions resulting from a high error frequency during the replication of the viral RNA [14], genetic changes in polioviruses can also occur by molecular genomic rearrangement during virus replication [15]. Poliovirus genomic rearrangement frequently takes place through homologous RNA recombination and mainly in the nonstructural coding regions of the viral genome. The frequency of recombination is about 2%, 53%, and 79% of poliovirus type 1, 2 and 3, respectively, and shows that the frequency depends strongly on the serotype of polioviruses [16]. Most crossover sites of the type 2 recombinants (S2/S1 and S2/S3 recombinants) lie in the *P3* coding region, and most crossover sites of type 3 recombinants (S3/S1 and S3/S2 recombinants) are located in the *P2* coding region [15], [17]. On the other hand, the crossover sites of very few type 3 recombinants (all were S3/S2 recombinants) [18], [19], [20], [21] and only 1 type 2 recombinant (S2/S3 recombinant) [5], are located in the *P1* coding region.

In this study, we describe 10 different natural type 3/type 2 capsid-recombinant polioviruses isolated from nine acute flaccid paralysis (AFP) case patients and one healthy vaccinee during the virological surveillance period 2001–2008 in China. Primary characterization of these isolates revealed that the first crossover sites of these 10 isolates were all in the *VP1* coding region. This observation led us to study the primary structure of the crossover sites and the genetic and phenotypic properties of these poliovirus chimeras.

## Results

### Primary characterization of the virus isolates

All 10 virus isolates were completely neutralized with polyclonal antisera specific for type 3 but could not be neutralized with antisera for type 2. Thus, they were all identified as type 3 polioviruses. Intratypic differentiation (ITD) tests were performed by two different methods. Polymerase chain reaction-restriction fragment length polymorphism (PCR-RFLP) ITD tests revealed typical Sabin 3 restriction patterns except for one strain (CHN6356) that showed an atypical non Sabin-like (NSL) pattern. In the enzyme-linked immunosorbent assay (ELISA) ITD tests, four isolates were identified as Sabin-like (SL), and two isolates (CHN5275 and CHN6053) were identified as double reactive virus (DRV), which indicated that they reacted with both Sabin 2-specific and type 2 wild poliovirus-specific cross-absorbed rabbit antisera. The results of ELISA ITD were not available for another four virus isolates due to the change of the ITD testing algorithm used in the laboratory (Table 1).

**Table 1:** Primary characterization of natural VP1 capsid recombinants isolated from AFP case patients and a healthy child.

Virus isolates	Accession numbers	Source*	Age(yr)/Sex	OPV history	Dates of			Intratypic differentiation †	
					Last OPV	Onset	Sampling	PCR-RFLP	ELISA
CHN5275	FJ859183	AFP case	0.8/F	3	11-Jun-2001	8-Aug-2001	14-Aug-2001	SL	DRV
CHN6053	FJ859184	AFP case	1.6/M	3	Unknown	28-Jan-2002	26-Feb-2002	SL	DRV
CHN6060	FJ859185	AFP case	0.7/M	1	5-Dec-2001	24-Jan-2002	4-Feb-2002	SL	SL
CHN6213	FJ859186	AFP case	0.8/M	1	Unknown	20-May-2002	26-May-2002	SL	SL
CHN6218	FJ859187	AFP case	1.5/M	1	5-Dec-2001	29-Jun-2002	3-Jul-2002	SL	SL
CHN6356	FJ859188	AFP case	0.4/M	2	15-Aug-2002	6-Sep-2002	10-Sep-2002	NSL	SL
CHN11144	FJ859189	AFP case	0.4/F	1	16-Jun-2007	17-Jul-2007	27-Jul-2007	SL	ND
CHN11185h	FJ859190	Healthy vaccinee	1.0/M	1	Unknown	—	1-Apr-2007	SL	ND
CHN12092	FJ859191	AFP case	3.2/M	3	16-Nov-2005	9-Sep-2008	9-Sep-2008	SL	ND
CHN12121	FJ859192	AFP case	0.3/M	1	1-Jul-2008	15-Aug-2008	22-Aug-2008	SL	ND

*AFP: Acute flaccid paralysis.

† SL: Sabin like; NSL: non Sabin-like; DRV: Double Reactive Virus; ND: not done.

**Figure 1:**
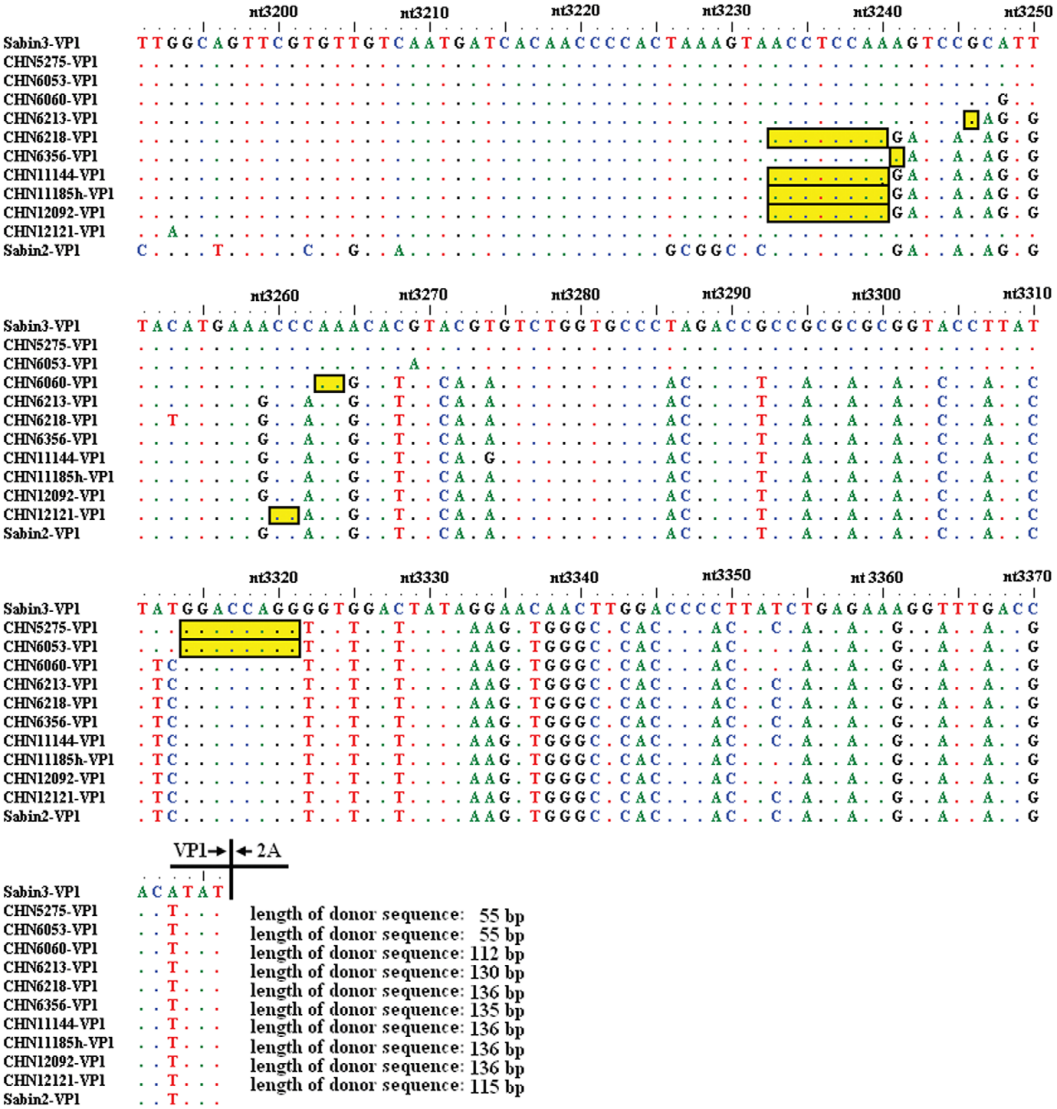
Nucleotide acid alignment of putative parental strains (P2/Sabin strain and P3/Sabin strain) and 10 type 3/type 2 intertypic VP1 capsid recombinants. The yellow open rectangles indicated possible crossover sites.

The entire *VP1* coding sequences of 10 poliovirus isolates revealed their uncommon genomic intertypic (type 3/type 2) recombinants with crossover sites within the *VP1* capsid-coding region. The last 55 to 136 nucleotides at the 3′ end of the *VP1* coding region were found to have high similarity (0–1 nucleotide substitutions) to the Sabin 2 strain, while the rest of the *VP1* coding region had high similarity to the Sabin 3 strain (1–4 nucleotide substitutions, and 99.37–99.87%), which showed their vaccine-related genomic features and revealed that their crossover sites were all located in the *VP1* coding region (Fig. 1).

### Reversions of important neurovirulence determination sites

Complete genomic sequencing of 10 natural type 3/type 2 intertypic vaccine-related poliovirus capsid recombinants with the first crossover sites within the *VP1* coding region (hereafter, *VP1* capsid recombinants) showed that their genomes were collinear with that of the Sabin 3 strain and that several mutations were scattered throughout the genomes. The nucleotide substitutions that had been identified as the principal determinants of the attenuated phenotype of the Sabin 3 strain had reverted through a U-to-C transition at nt472 in the *5*′*-UTR* region and a C-to-U transition at nt2493 in the *VP1* coding region, leading to a Thr-to-Ile amino acid substitution of residue 6 of VP1 in all 10 isolates. Strain CHN11185h also exhibited a transition U-to-C reversion at nt2034 in the *VP3* coding region, another important neurovirulence determinant of type 3 polioviruses, which leads to a Phe-to-Ser amino acid substitution of residue 91 of VP3 [22], [23] (Table 2).

**Table 2:** Genetic and phenotypic characterizations of natural VP1 capsid recombinants.

Virus isolate	Nucleotide and amino acid of neurovirulence determinants					Recombination pattern	Evolution of the viruses*	
	*5′-UTR*	*VP3*		*VP1*				
	nt472	nt2034	aa91	nt2493	aa6		*Ks* (days)	*Kt* (days)
P3/Sabin	***U***	***U***	***Phe***	***C***	***Thr***	—	—	—
CHN5275	C	***U***	***Phe***	U	Ile	S3/S2/S3	0.34 (38 d)	0.27 (88 d)
CHN6053	C	***U***	***Phe***	U	Ile	S3/S2/S3	0.56 (63 d)	0.39 (127 d)
CHN6060	C	***U***	***Phe***	U	Ile	S3/S2/S3	0.47 (53 d)	0.24 (79 d)
CHN6213	C	***U***	***Phe***	U	Ile	S3/S2/S3	0.35 (39 d)	0.24 (79 d)
CHN6218	C	***U***	***Phe***	U	Ile	S3/S2/S3	0.46 (52 d)	0.24 (79 d)
CHN6356	C	***U***	***Phe***	U	Ile	S3/S2/S3	0.35 (39 d)	0.20 (65 d)
CHN11144	C	***U***	***Phe***	U	Ile	S3/S2/S1/S2/S1	0.58 (65 d)	0.36 (118 d)
CHN11185h	C	C	Ser	U	Ile	S3/S2/S3	1.07 (120 d)	0.36 (118 d)
CHN12092	C	***U***	***Phe***	U	Ile	S3/S2/S3	0.46 (52 d)	0.32 (105 d)
CHN12121	C	***U***	***Phe***	U	Ile	S3/S2	0.46 (52 d)	0.20 (65 d)
P3/Leon/37	C	C	Ser	U	Ile	—	—	—

Shaded area indicates the nucleotides and amino acids that were identical to the Sabin 3 strain, numbering according to the Sabin 3 strain (GenBank accession no: AY184221).

### Antigenic divergence of the VP1 capsid recombinants

The results of the ELISA ITD method showed that strains CHN5275 and CHN6053 were two antigenic variants of the Sabin 3 strain; however, all 10 isolates were completely neutralized with polyclonal antisera to type 3 poliovirus but not with type 2 poliovirus antisera, according to the standard procedure [24]. The amino acid sequences within or near the predicted neutralizing antigenic (NAg) sites [25] were aligned with 10 *VP1* capsid recombinants, Sabin 3 strain and its neurovirulent precursor (Leon/USA/1937), Sabin 2 strain, and some type 3 wild polioviruses circulating in different parts of the world during the period 1960–2004. There were four amino acid substitutions in the NAg sites among these VP1 capsid recombinants: One substitution was in NAg1 (strain CHN6356, VP1-98: Arg to Gln), the second in NAg2 (strain CHN11144, VP2-165: Ala to Thr), the third in NAg3a (strain CHN12092, VP3-59: Ser to Gly), and the fourth in NAg3b (strain CHN6053, VP3-78: Ser to Phe). In addition, the whole NAg3a (VP1-286 to VP1-290) in the VP1 coding region was completely replaced by Sabin 2-specific amino acid sequences (Fig. 1).

**Figure 2:**
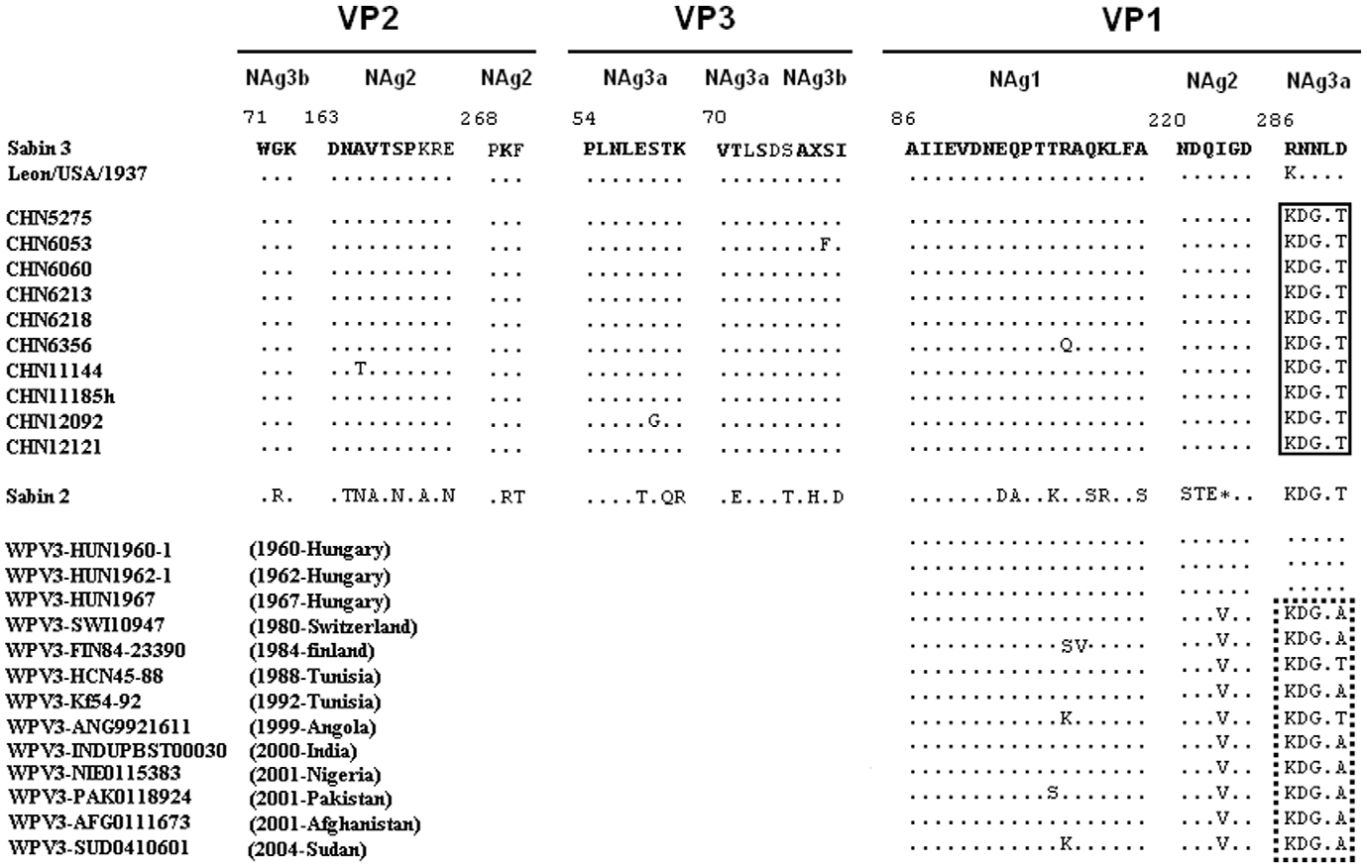
Alignment of amino acid residues of neutralizing antigenic (NAg) sites 1 (*VP1*: 88–106), 2 (*VP2*: 163–169; *VP2*: 268–270; *VP1*: 220–225), 3a (*VP3*: 54–61; *VP3*: 70–74; *VP1*: 286–291), and 3b (*VP2*: 71–73; *VP3*: 75–79) for Sabin 3, its parental strain Leon/USA/1937, *VP1* capsid recombinants, Sabin 2, and some wild-type polioviruses circulated worldwide after 1960 (GenBank accession numbers: EU918372, EU918375, EU918374, FJ914252, FJ842179, AM707037, AM707033, AY221236, AY189887, AY221231, AY221227, AY221224, and AY741367). The rectangle shows NAg3a displacement in VP1 from Sabin 3 to Sabin 2 due to the recombination. The dashed rectangle shows NAg3a of type 3 wild polioviruses circulated after 1980.

Interestingly, the NAg3a amino acid in the VP1 protein of type 3 wild polioviruses that were circulating throughout the world after 1980 were similar to that of the Sabin 2 strain, and the amino acid sequences were identical between VP1-286 and VP1-289. Type 3 wild polioviruses isolated from Angola and Tunisia also have the same residues of VP1-290, and type 3 wild polioviruses isolated from Nigeria, India, Pakistan, and Afghanistan, where wild polioviruses are still circulating, have a different residue at VP1-290 (from Thr to Ala). The neurovirulent precursor of Sabin 3 (Leon/USA/1937) also had a Lys residue at VP1-286 that was identical to that of the Sabin 2 strain (Fig. 2).

### Recombination features of the VP1 capsid recombinants

**Figure 3:**
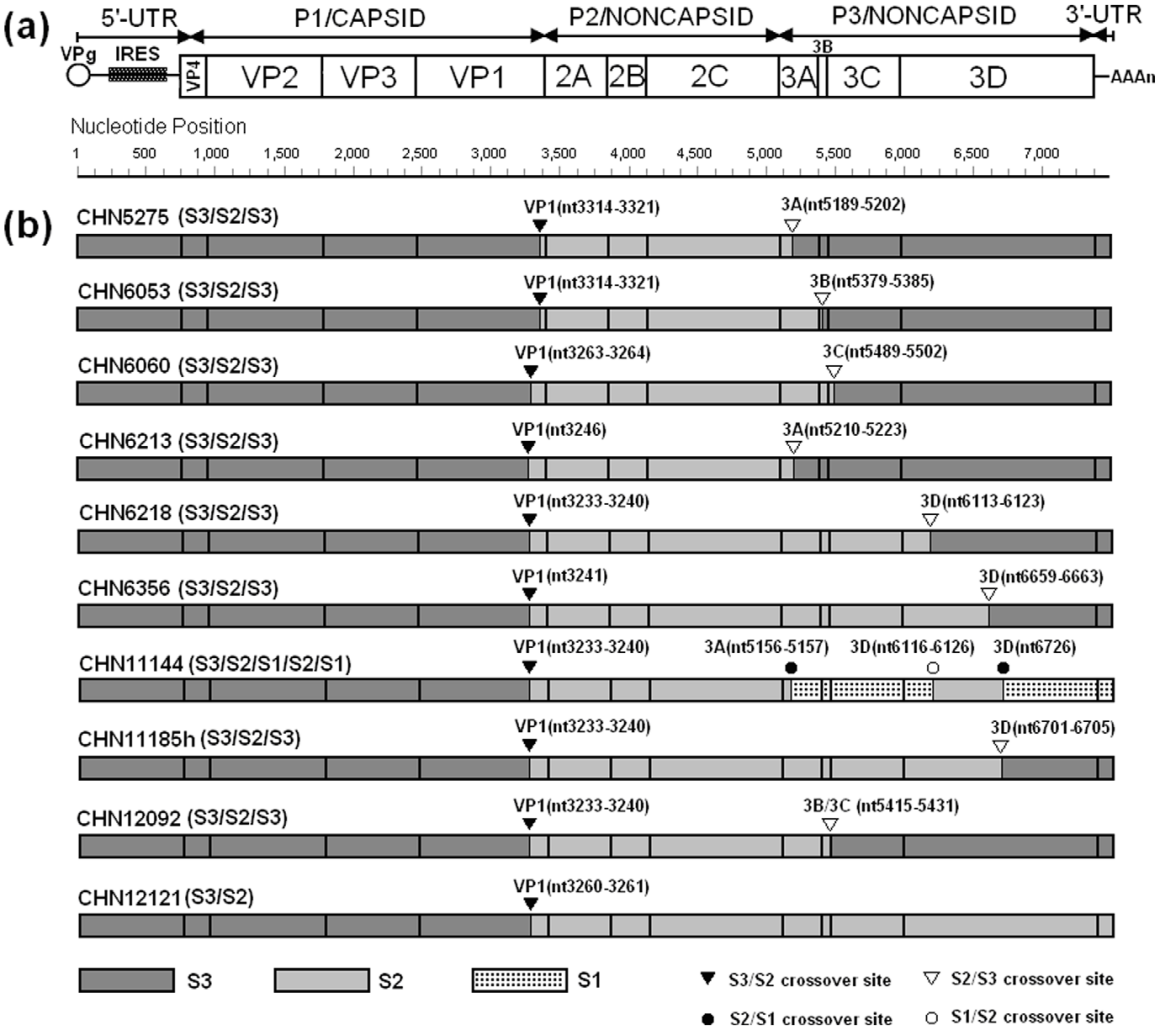
Schematics of the genomes for the 10 type 3/type 2 intertypic VP1 capsid recombinants. (a): Schematic genetic organization of Sabin 3 reference strain (GenBank accession number AY184221). The single open reading frame, flanked by *5′-UTR* and *3′-UT*R, is indicated by a rectangle. (b): Structures of 10 type 3/ type 2 Sabin intertypic capsid recombinants; type 1, type 2, and type 3 poliovirus sequences are indicated (abbreviated as S1, S2, and S3, respectively). Symbol ▾ indicates the location of S3/S2 crossover sites; symbol ▽ indicates the location of S2/S3 crossover sites; symbol • indicates the location of S2/S1 crossover sites; and symbol ○ indicates the location of S1/S2 crossover sites. The positions of crossover sites are indicated at the top of each of the symbols.

**Figure 4:**
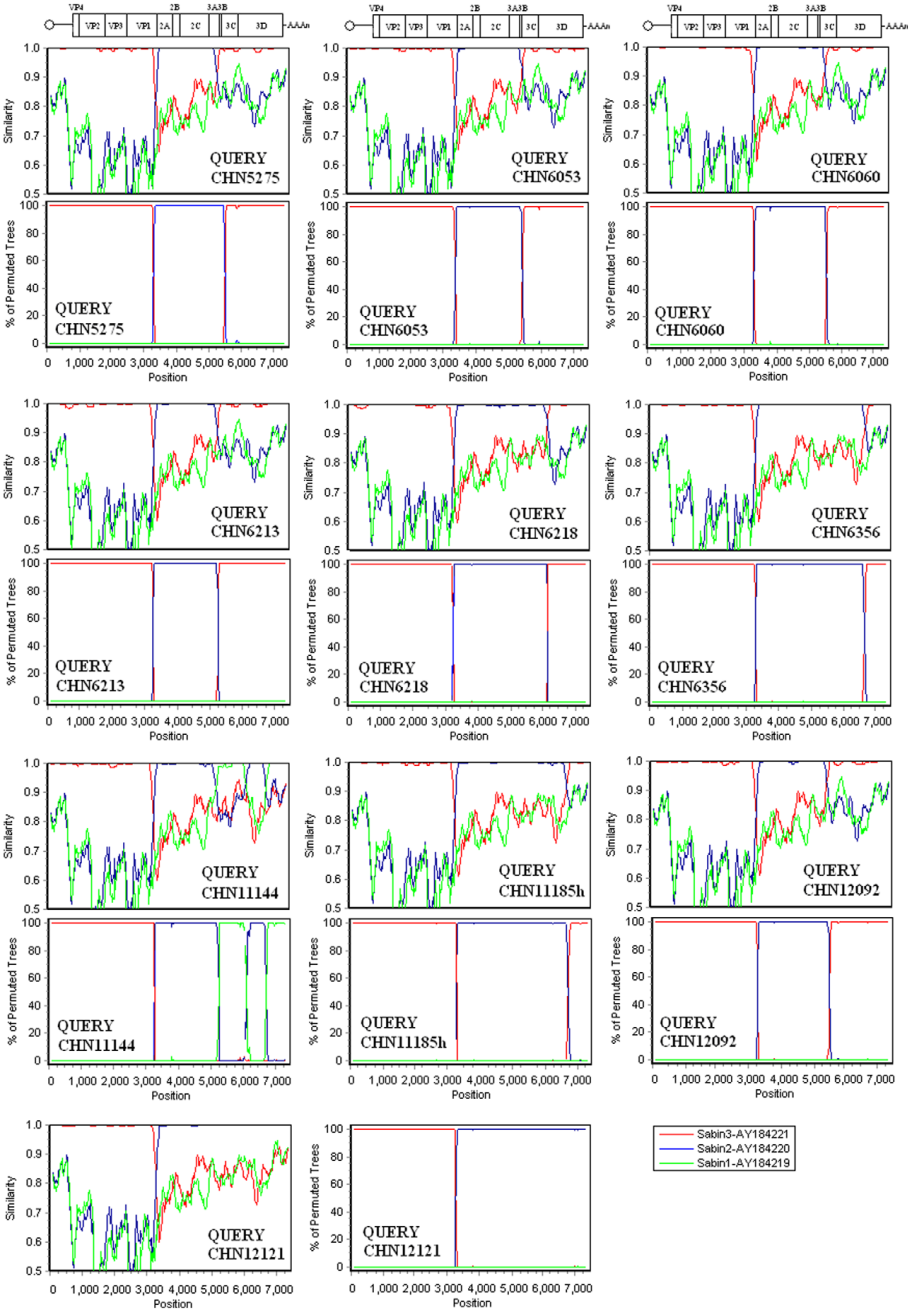
Similarity plot and bootscanning analysis of complete genomes of 10 type 3/type 2 intertypic VP1 capsid recombinants using a sliding window of 200 nt moving in 20-nt steps. For each plot, the names of viruses of the query sequence were indicated in the bottom right corner, and for each bootscanning analysis, the names of viruses of the query sequence were indicated in the bottom left corner.

Eight of the ten *VP1* capsid recombinants (strains CHN5275, CHN6053, CHN6060, CHN6213, CHN6218, CHN6356, CHN1185h, and CHN12092) were found to have recombination in two crossover sites: The 5′ part of the genome was the Sabin 3 sequence; the middle part was the Sabin 2 sequence; and the 3′ part was the Sabin 3 sequence (S3/S2/S3). The first crossover sites were located in the *VP1* coding region, and the second crossover sites were in the *P3* region but at different positions, which could be identified in all four nonstructural coding regions *(3A*, *3B^VPg^*, *3C* or *3D^Pol^)* (Fig. 3 and Fig. 4).

Strain CHN12121 was simpler in that it had only one crossover site located between nt3260 and nt3261 in the *VP1* coding region; the 5′ part of the genome was the Sabin 3 sequence, and the 3′ part was the Sabin 2 sequence (S3/S2) (Fig. 3 and Fig. 4).

Strain CHN11144 was a rare and complicated multi-recombinant poliovirus, and its genomic organization was characterized as a S3/S2/S1/S2/S1 tetra-recombinant. The complete genomic sequences revealed the presence of four crossover sites. The first was from a S3/S2 recombination event with the crossover site located between nt3233 and nt3240 in the *VP1* coding region, while the second crossover site was from a S2/S1 recombination event located between nt5156 and nt5157 in the *3A* coding region. The third and the fourth crossover sites were from S1/S2 and S2/S1 recombination events, with the two crossover sites located in nt6116–6126 and nt6726 in the *3D* coding region, respectively (Fig. 3 and Fig. 4).

Although the numbers and positions of crossover sites were different among the 10 *VP1* capsid recombinants, they all had the first crossover sites located in the *VP1* coding region. The lengths between the crossover sites and the 3′ end of the *VP1* coding region ranged from 55 to 136 nucleotides, and the crossover sites of strains CHN6218, CHN11144, CHN11185h, and CHN12092 were located between nt3233 and nt3240, which produced the longest lengths of donor Sabin 2 genome sequences in the literature for this type of poliovirus recombination by now (numbering refers to the Sabin 3 genomic sequences, GenBank accession number AY184221) (Fig. 1).

Although different lengths of nucleotides (55 to 136 nucleotides) were introduced in *VP1* coding region in each of the recombinant isolates, four amino acid changes (VP1-286: Arg to Lys, VP1-287: Asn to Asp, VP1-288: Asn to Gly, and VP1-290: Asp to Thr) in Nag3a were introduced among these ten VP1 capsid recombinants, which lead the whole NAg3a of these ten recombinants were completely replaced by Sabin 2-specific amino acid sequences.

### Temperature sensitivity

All 10 VP1 capsid recombinants were compared to P3/Sabin strain as regards replication capacity at an elevated temperature (40°C), and showed different results of temperature sensitivities. P3/Sabin was temperature sensitive as expected with titer reduction of more than 2 logarithms at 36°C /40°C, and five VP1 capsid recombinants (CHN5275, CHN6060, CHN6218, CHN6356 and CHN12121) appeared similar results as P3/Sabin strain, while the property of temperature sensitivity of other five VP1 capsid recombinants showed a definitely lesser effect (titers reduced less than 2 logarithms at 36°C /40°C), this means that replication efficiency of them remains the same even at elevated temperatures (Table 3).

**Table 3:** Temperature sensitivity of 10 natural type 3/type 2 capsid-recombinant polioviruses.

	Growth temperature and virus titer					
	36°C		40°C		Log titer reduction 36°C/40°C	
Virus strain	8 h p.i.	24 h p.i.	8 h p.i.	24 h p.i.	8 h p.i.	24 h p.i.
P3/Sabin	6.500	8.250	3.250	3.750	3.250	4.500
CHN5275	8.500	8.875	7.000	6.750	1.500	2.125
CHN6053	7.625	8.250	7.500	7.500	0.125	0.750
CHN6060	8.375	8.875	6.875	6.625	1.500	2.250
CHN6213	7.875	8.125	6.750	6.750	1.125	1.375
CHN6218	7.750	7.875	6.125	5.625	1.625	2.250
CHN6356	8.000	7.875	6.125	5.625	1.875	2.250
CHN11144	8.250	8.750	7.125	7.375	1.125	1.375
CHN11185h	8.500	9.000	7.125	7.125	1.375	1.875
CHN12092	8.500	8.500	7.250	7.125	1.250	1.375
CHN12121	8.000	8.375	5.750	5.125	2.250	3.250

*Ks: synonymous substitutions, *Kt*: total substitutions.

### Estimation of the evolution time of the VP1 capsid recombinants

The approximate evolution times of the polioviruses were estimated from the P1/capsid sequence differences between *VP1* capsid recombinants and the Sabin 3 reference strain. The corrected proportion of synonymous substitutions (*Ks*) was 0.33–1.07% of synonymous sites in the P1/capsid region (not including the donor recombinant sequences), and, of the total substitutions (*Kt*), was 0.20–0.39%. Under the assumption of constant nucleotide substitution rates of 3.2% synonymous substitutions per synonymous sites per year and 1.1% total substitutions per site per year in the *P1/capsid* region [26], we estimated that the ages of the *VP1* capsid recombinants were 38–120 days (from the *Ks* estimate) and 65–127 days (from the *Kt* estimate), respectively.

## Discussion

Poliovirus is one of the first recognized viruses to undergo recombination [27]. Recombination has been found frequently in wild polioviruses, VDPVs, and vaccine-related polioviruses [5], [28], [29]. Usually, the first crossover site is in the *P2* or *P3* nonstructural region and often within the *3D* coding region [15]. Natural intertypic capsid recombination between Sabin strains is a relatively rare phenomenon, possibly due to structural constraints that maintain the integrity of the capsid shell. The integrity of the capsid region of poliovirus seems to be very important for propagation of the viruses themselves. Some *in vitro* experiments have shown that if the crossover site is located in the capsid-coding region, the intertypic recombinant chimera will be nonviable [30]; in addition, some other experiments have shown that, compared to its parental strains, the intertypic recombinant chimera with the crossover site in the capsid-coding region is unstable [31].

Some similar intertypic recombinant chimera have been reported previously [18], [19], [20], [21], but to the best of our knowledge, 136 nucleotides insert is the longest donor sequence that has been reported, so the longest poliovirus type 2 insert in the *VP1* coding region of poliovirus capsid recombinant has been reported herein.

Although the first crossover sites were different from each other among the 10 *VP1* capsid recombinants, they all gained type 2 NAg3a amino acids in the VP1 structure protein in the Sabin 3 background. Although the replacement of NAg3a in these recombinant viruses could result in partial antigenic changes, as shown before [18], [20], the overall type 3 antigenic structure was not significantly altered since all virus isolates were readily neutralized by type 3 polyclonal serum. This meant that NAg3a may have altered some of the type 3-specific antigenic properties but that Sabin 3 had not acquired any type 2-specific characterizations, possibly based on the fact that the inserted type 2 NAg3a is located on the surface of the virion and is implicated in receptor binding, allowing more freedom for aberrant folding [32].

The NAg3a amino acid of the Sabin 3 strain seems atypical; other type 3 wild poliovirus isolates that have circulated worldwide in recent years (after 1980) have sequences of NAg3a more like the Sabin 2 strain. But the type 3 wild poliovirus isolates from earlier year (before 1967) still have sequences of NAg3a more like the Sabin 3 strain. The possibility exist that recent type 3 wild poliovirus isolates may be a recombinant having NAg3a sequences derived from another strain during between 1967 and 1980. It seems likely, but is hard to prove, that perhaps the type 3/type 2 recombination events in the 3′ end of *VP1* coding region may have a higher fitness.

The results of age estimation guided us towards the reconstruction of the potential histories of these clinical isolates. OPV strains had replicated in the human gut for 38–120 days (from the Ks estimate) and for 65–127 days (from the Kt estimate) after the initial OPV doses were given. Based on the fact that type 2 and type 3 vaccine viruses can replicate and be excreted by immunocompetent vaccinees for about 3 months after vaccination [33] and considering the small dispersion of ages, the possibility that each particular recombination event occurred in the person from whose stool specimen the virus was isolated cannot be excluded. During the short time period, nucleotide substitutions and genetic exchanges were selected very quickly, probably as a response to different selective advantages, to increase the fitness of the Sabin strains for replication in the human gut by preventing the accumulation of harmful mutations.

The limitation with the method of the estimation of evolution used in this study is that this approach assumes a fixed substitution rate and multiplies it by the estimated number of nucleotide substitutions (total substitutions or synonymous substitutions), but in fact, the actual amount of evolution (number of substitutions) mainly depends on the virus effective population size inside the host and the frequency of replication. Additionally, the recombination events would lead to a change in the selection pressure, and most likely, there would be an increase in selection resulting in an increased evolutionary rate, meaning that the ages of the VP1 capsid recombinants may have been overestimated. This is most obvious in the case of CHN11144, whose estimated ages (65 days from the *Ks* estimate or 118 days from the *Kt* estimate) are greater than the time from administration of OPV to sampling (41 days).

All the children had one or more OPV before sampling, and the intervals between the dates of administration of last OPV and sampling are from 26 days to 1027 days, which indicated not all natural VP1 capsid recombinants directly derived from the OPV strains they received, most likely, some children were affected by them from environment. There was evidence that, like other vaccine-like polioviruses, VP1 capsid recombinants could survive and be detected from the environmental sewage [21].

Genetic attenuation usually decreases viral fitness, and all the *VP1* capsid recombinants had nucleotide substitutions of U-to-C at nt472 in the *5′-UTR* region and a transition C-to-U at nt2493, which resulted in a Thr-to-Ile substitution of residue 6 of VP1, and these were direct reversions to the neurovirulent precursor of Sabin 3, Leon/USA/1937. These two nucleotide substitutions have also been frequently found in type 3 VDPV strains, including 2002 VDPV isolates from sewage in Estonia [34], 2006 iVDPV isolates from an Iranian child [35], and in the Sabin 3-related polioviruses [36], which may indicate that these “hot spots” faced intensive selective pressures as the OPV strain replicated in the human gut.

Five of the VP1 capsid recombinants (CHN5275, CHN6060, CHN6218, CHN6356 and CHN12121) did not lose the temperature-sensitive phenotype of parent P3/Sabin strain. This result differed to three previously reported capsid recombinants which had almost entirely lost the temperature sensitivity [18], [19], [20], but was similar to a recent report [21], and other five VP1 capsid recombinants in this study had almost entirely lost the temperature sensitive phenotype. It had been reported that the temperature sensitivity phenotype of type 3 poliovirus is mainly attributable to a difference in residue 91 of the VP3 capsid protein [37], but this amino acid substitution could not be found in the VP1 capsid recombinants in this study except strain CHN11185h (Table 2), and this might explain the preservation of temperature-sensitive phenotype of the viruses. Besides residue 91 in VP3 capsid region, 11 other amino acid substitutions have been suggested to be related to temperature sensitivity, four in VP1 region (residues 34, 54, 132 and 263), four in VP2 region (residues146, 200, 215 and 265), and three in VP3 region (residues 108, 175 and 178) [37]. Among these, the substitution Ala to Thr or Ala to Val at position 54 in the VP1 capsid region were found in strains CHN5275, CHN6053 and CHN12092.

In conclusion, 10 natural type 3/type 2 intertypic *VP1* capsid-recombinant polioviruses, in which the first crossover sites were found to be in the *VP1* coding region, were isolated and characterized. In spite of the complete replacement of NAg3a by type 2-specific amino acids, the serotypes of the recombinants were not altered, and they were totally neutralized by polyclonal type 3 antisera but not at all by type 2 antisera. NAg3a of the Sabin 3 strain seems atypical; other wild-type poliovirus isolates that have circulated in recent years have sequences of NAg3a more like the Sabin 2 strain. It is possible that recent type 3 wild poliovirus isolates may be a recombinant having NAg3a sequences derived from another strain during between 1967 and 1980, and the type 3/type 2 recombination events in the 3′ end of the *VP1* coding region may result in a higher fitness.

## Materials and Methods

### Stool specimens

This study did not involve human participants and did not contain human experimentation, the only used material is stool samples collected from the AFP case patients for the purpose of public health initiated by World Health Organization and Chinese Ministry of Health, and the written informed consents from all participants involved in this study were obtained for the use of their stool samples. This study has been approved by the second session of Ethics Review Committee in Chinese Centre for Disease Control and Prevention.

10 type 3 polioviruses were isolated from nine AFP case patients (from Jiangxi, Henan, Yunnan, Guangdong, Gansu, Guizhou, Hubei, and Hebei provinces) and a healthy vaccinee (from Xizang Autonomous Region) during the period 2001–2008 in China. All the AFP case patients were less than 1.6 years old when they presented with symptoms, except for one patient from Hubei province (a 3.2-year-old boy). All of the children with AFP had at least one dose of OPV after birth; however, it was unknown when the healthy vaccinee from the Xizang Autonomous Region had received a dose of OPV because a written record of this could not be located. None of the patients with AFP showed signs of immunodeficiency at the time of presentation. Two stool specimens were collected from each of the nine AFP case patients at 24 hours apart within 14 days after onset of symptoms. One stool specimen was collected from the healthy vaccinee during an epidemiological survey of OPV coverage in 2007 (Table 1).

### Viral isolation and primary identification

RD (Human rhabdomyosarcoma) and L20B (mouse L cell expressing the human poliovirus receptor) cell lines were used to isolate viruses from the stool specimens by standard procedures [24]. All L20B-positive isolates were identified by a micro-neutralization test with poliovirus type-specific rabbit polyclonal antisera (National Institute for Public Health and the Environment [RIVM], Bilthoven, The Netherlands) [24]. Two ITD methods, both targeting the *VP1* coding region, were used to investigate the wild or vaccine origin of the poliovirus isolates. First, we employed the PCR-RFLP method, which was based on the genetic properties of the polioviruses [38]; this method identified a poliovirus as either typical SL or atypical NSL. We then applied the ELISA method, which was based on the antigenic properties of the polioviruses [39]; using this method, poliovirus isolates were classified into one of the following four different groups formed on the basis of antigenic properties: SL, NSL, DRV, and nonreactive virus (NRV).

### Viral RNA extraction and reverse transcription

Viral RNAs were extracted from the viral isolates using a QIAamp Viral RNA Mini Kit (Qiagen, Valencia, CA, USA) and stored at −80°C for further use. 1µl (200U) SuperScript II ribonuclease H- reverse transcriptase (invitrogen, USA) was used to produce single stranded cDNA from 5µl of each purified viral RNA. The cDNA syntheses were primed by 7500A and Q8 (Table 4), respectively, and performed at 42°C for 2h, followed at 60°C for 15 min to inactivate the enzyme. Finally, RNA in an RNA:DNA hybrid was specifically degraded with 1µl ribonuclease H (Promega, USA) at 37°C for 30 min.

**Table 4:** PCR and sequencing primers.

Primer	Nucleotide position (nt)	Primer sequence (5-3)	Orientation	Reference
0001S48^a^		GGGGACAAGTTTGTACAAAAAAGCAGGCTTTAAAACAGCTCTGGGGTT	Forward	[6]
EV/PCR-2	449–473	TCCGGCCCCTGAATGCGGCTAATCC	Forward	[42]
EV/PCR-1	537–562	ACACGGACACCCAAAGTAGTCGGTCC	Reverse	[42]
1275S	1275–1294	ACGTGCAGTGTAATGCATCC	Forward	This study
1511A	1492–1511	TTCCCAGTAACACCCCACAT	Reverse	This study
1949S	1949–1969	AACACAATGGACATGGTATAG	Forward	This study
2224A	2205–2224	ACATGTGTGCCCAACATAGC	Reverse	This study
Y7^a^	2397–2419	GGTTTTGTGTCAGCGTGTAATGA	Forward	[43]
2873A	2853–2873	GAATTCCATGTCAAATCTAGA	Reverse	This study
3368S	3368–3389	AACGACTTATGGATTTGGACAC	Forward	This study
Q8^a^	3477–3496	AAGAGGTCTCTRTTCCACAT	Reverse	[43]
4443S	4443–4465	AAYTACATACAGTTCAAGAGCAA	Forward	[5]
4489A	4468–4489	AAACATACTGGCTCAATACGGT	Reverse	[6]
5076S	5076–5097	GGTAATTGCATGGAAGCTCTAT	Forward	This study
5274A	5253–5274	GGTTGATGTTCCTCTCTGTTTG	Reverse	This study
5904S	5904–5929	GGGATGCATGTYGGIGGGAACGGTTC	Forward	This study
6097A	6077–6097	GGTTCCTTAACTCCTTCAAAC	Reverse	[6]
6914S	6914–6934	CTACAAGGGCCTAGATTTAGA	Forward	[5]
6970A	6951–6970	ATTACATCATCACCATAGGC	Reverse	This study
7500A^a^		GGGGACCACTTTGTACAAGAAAGCTGGG(T)_24_	Reverse	[6]

a The primer pairs Y7/7500A and 0010S48/Q8 are suggested for long distant PCR. The expected amplicons from these are 5.28 kb and 3.57 kb, respectively.

### Full-length genome amplification

Two long-distant PCR amplifications (for one virus) were performed by using the TaqPlus Precision PCR system (Stratagene, USA), which consists of a blend of Stratagene cloned Pfu DNA polymerase (proof reading) and Taq2000 DNA polymerase (non-proof reading). Reactions contained 5µl of cDNA (see above), 0.1mM of each dNTP, 10µl of TaqPlus buffer, 1.0 ng/µl of a forward (0001S48 or Y7) and reverse (Q8 or 7500A) primer (Table 4), and 5 units TaqPlus enzyme in a 100µl reaction. The amplification was carried out by 30 times cycling through temperature levels of 94°C (30 s), 60°C (30 s), and 72°C (6 min), and followed by another two temperature levels of 94°C (1min) and 72°C (20 min).

### Nucleic acid sequencing

Two long-distant PCR products (for one virus) were purified using a QIAquick Gel Extraction Kit (Qiagen, Valencia, CA, USA). Cycle sequencing reactions were carried out using the version 3.0 of the BigDye terminator chemistry (Applied Biosystems), using the primers listed in Table 4. Sequencing was performed in both directions using an ABI PRISM 3100 Genetic Analyzer (Applied Biosystems), and every nucleotide position was sequenced at least once from each strand. 5′ segment sequences were determined by using the 5′ rapid amplification of cDNA ends core set (Takara Biomedicals) according to the manufacturer's instructions.

### Location of the crossover sites

The sequences of the isolates were aligned with the reference strains by using the MEGA program v4.0 (Sudhir Kumar, Arizona State University, Arizona, USA) [40]; the resulting reference strain sequences were found to be GenBank sequences under the accession numbers AY184219, AY184220 and AY184221 for Sabin 1, Sabin 2 and Sabin 3, respectively. Plots of nucleotide similarity were created using the SimPlot program v3.5.1 (Stuart Ray, Johns Hopkins University, Baltimore, Maryland, USA) [41]. The crossover sites were identified as located between the last nucleotide, differentiating the clinical sequence from the 3′ partner reference sequences, and the first nucleotide, differentiating the clinical sequence from the 5′ partner reference sequence.

### Assay for temperature sensitivity

Temperature sensitivities of 10 natural type 3/type 2 capsid-recombinant polioviruses were assayed on monolayer RD cells in 24-well plates as described before [18]. Briefly, the 24-well plates were inoculated with 50 µl of undiluted virus stocks (P3/Sabin and 10 VP1 capsid-recombinants). And two different incubators were used; the temperature of one incubator was adjusted to 36°C (optimal temperature for virus propagation), while the temperature of the other incubator was adjusted at 40°C (supraoptimal temperature for virus propagation). After absorption at 36°C or at 40°C for 1 h, the unabsorbed virus inoculums was removed, 100 µl of maintenance medium was added to each well and the plates were continually incubated at 36°C or at 40°C, separately. After 8 h and 24 h post-infection, the plates were harvested, and the cell culture infectious dose 50% (CCID_50_) was calculated by the end-point dilution method on monolayer RD cells in 96-well plates at 36°C. Virus isolate showing more than 2 logarithms reduction of the titers at different temperatures was considered to be temperature sensitive.

### Estimation of the date of the initiating OPV dose

The date of the initiating OPV dose for each patient was estimated from the *Ks* (synonymous substitutions per synonymous site) and *Kt* (all the substitutions per site) values by assuming evolution rates of 0.032 synonymous substitutions per synonymous site per year and 0.011 total substitutions per site per year [26].

### Nucleotide sequence accession numbers

The complete genomic sequences of 10 natural type 3/type 2 capsid-recombinant polioviruses described in this study were deposited in the GenBank database under the accession numbers FJ859183 to FJ859192.
